# Supplementary material for: Activity-dependent expression of Channelrhodopsin at neuronal synapses
Source: Nat Commun. 2017 Nov 20;8:1629. doi: 10.1038/s41467-017-01699-7 (PMC5696361; doi:10.1038/s41467-017-01699-7)
Supplement: Supplementary file 1 — Supplementary Information [file 41467_2017_1699_MOESM1_ESM.pdf]

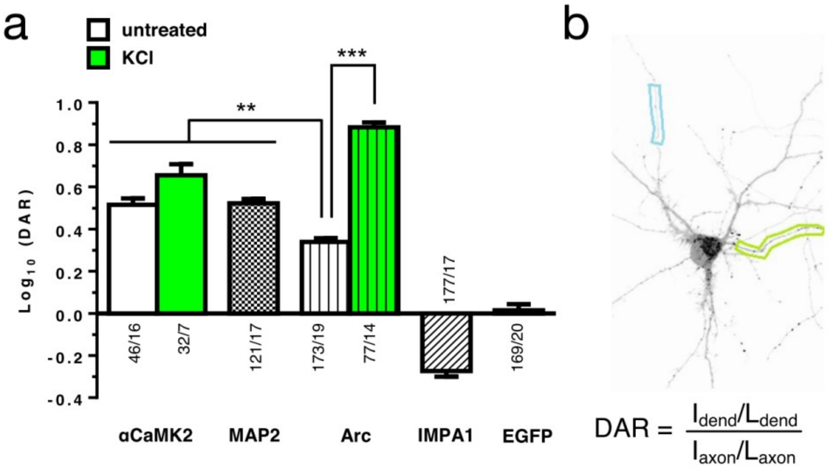

Supplementary Figure 1 *Arc* DTE drives activity-dependent dendritic expression of palmitoyl-Cherry. For its ability to determine low mRNA translation in basal conditions and strong translation upon depolarization, *Arc* was the best candidate among the DTEs we tested. (**a**) Plot of dendrite-to-axon ratio (DAR) of protein expression. When *Arc* DTE is present, Cherry is enriched in dendrites relative to EGFP or other soma-translated proteins. *Arc* DAR is significantly lower than alphaCaMKII and MAP2 DTEs in untreated neurons, but 60 minutes KCl 10mM significantly increases *Arc* DAR. alphaCaMKII DTE also increases DAR upon KCl stimulation but the effect is less prominent than for *Arc*. As control, we included the IMPA1-derived ATE. Numbers indicate the number of dendrites/neurons analyzed (**b**) Example illustrating DAR calculation. DAR is defined as the ratio of the Cherry intensity (I) per length (L) in the dendrite divided by the corresponding intensity per length in the axon. Light blue region corresponds to a dendrite region, green one to axon. $^{**}P<0.01$ and $^{***}P<0.001$ one-way ANOVA, Bonferroni comparison of means. Bars are mean±s.e.m. N and replicate numbers for all figures are listed in Supplementary Table 1.

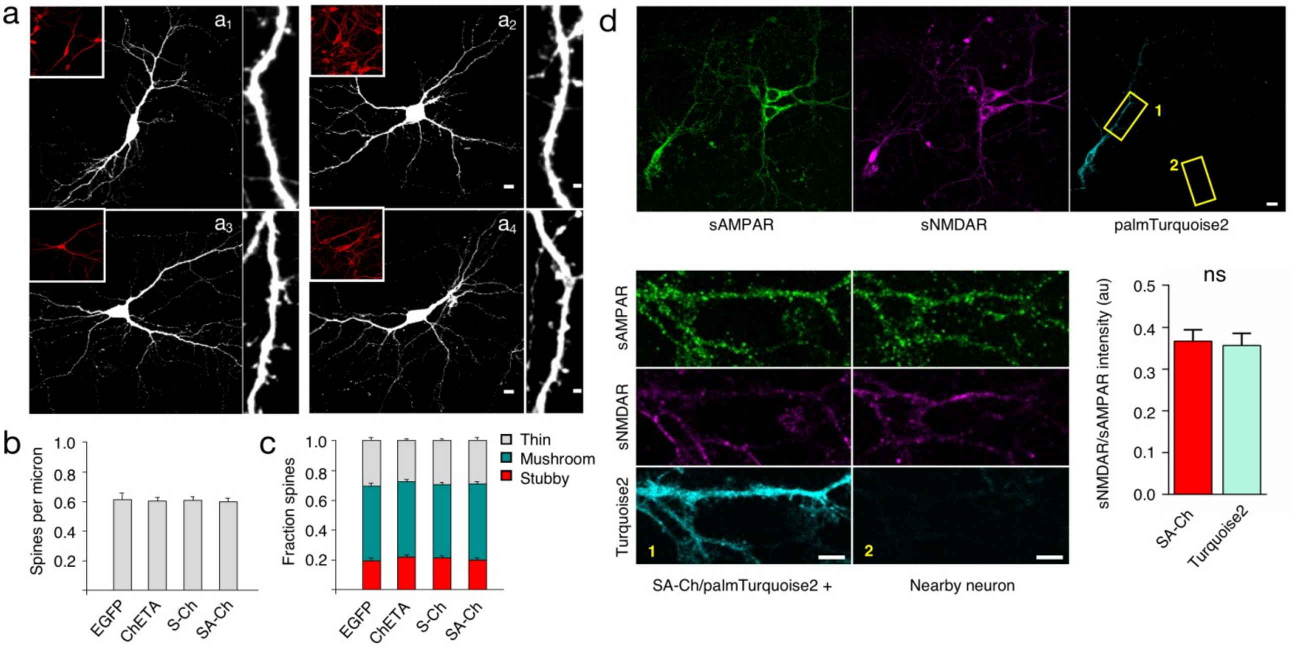

Supplementary Figure 2 SA-Ch expression does not alter neuron morphology and spine density. (**a**) Representative neurons transfected with (a_1) EGFP alone, (a_2) ChETA and EGFP, (a_3) S-Ch and EGFP, and (a_4) SA-Ch and EGFP. Inset (red) MAP2 immunofluorescence. On the right of each neuron, a magnification of the dendritic arbour. Scale bars: main image 10 µm, magnification 2 µm. (**b**) Quantification of average number of dendritic spines per micron. Results are not significantly different at the 0.05 level, one-way ANOVA, Bonferroni comparison of means. (**c**) SA-Ch does not alter spine morphology. Quantification of spine class frequency (stubby, mushroom, thin) for the four groups. Results are not significantly different at the 0.05 level, one-way ANOVA, Bonferroni comparison of means. (**d**) SA-Ch expression does not alter the ratio of surface NMDAR/AMPAR (sNMDAR and sAMPAR). Top, representative image of a neuron expressing SA-Ch/palmitoyl-Turquoise2 stained for superficial AMPAR and NMDAR. Bottom, magnification of dendrites from the SA-Ch/palmitoyl-Turquoise2 positive neuron (Region 1) and from a nearby neuron (Region 2). Scale bars: main image 10 µm, magnifications 5 µm. Quantification of the surface NMDAR/AMPAR ratio for neurons transfected with SA-Ch/palmitoyl-Turquoise2 (SA-Ch sample) or palmitoyl-Turquoise2 only (Turquoise sample). Difference is not significative (ns, P=0.61, Student's t test, two-tailed). Bars are means±s.e.m.

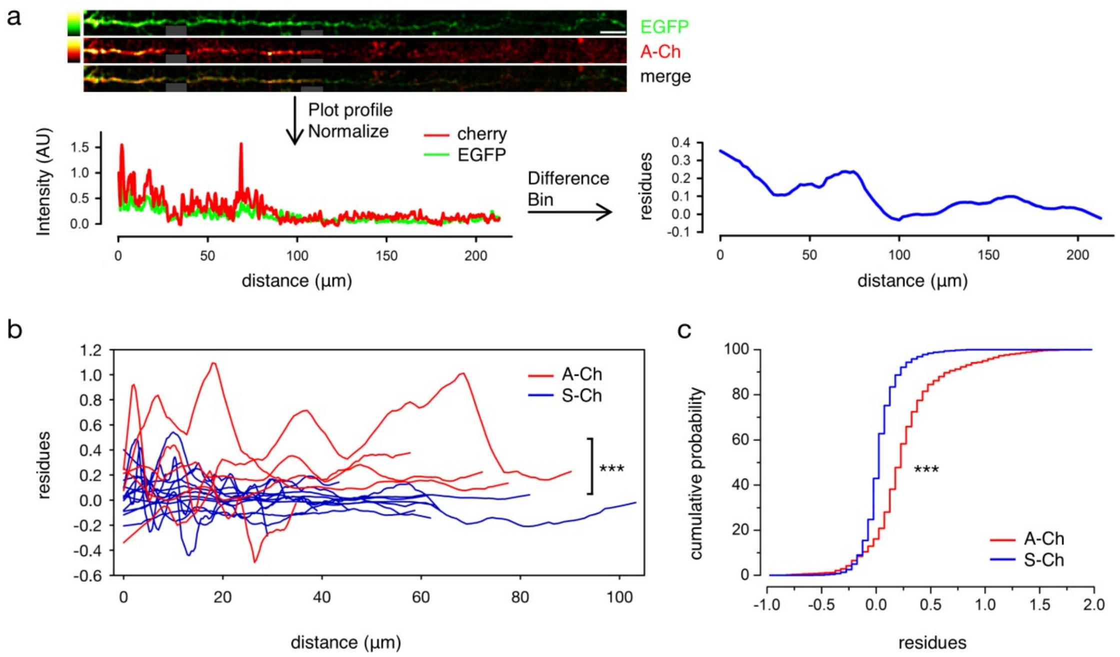

Supplementary Figure 3 *Arc* sequences increase ChETA-Cherry intensity following BDNF-dependent L-LTP and activation of translation. (**a**) Outline of procedure: EGFP and Cherry intensities along dendrites are plotted and normalized to the value 10 µm away from the centre of soma; the difference is plotted as difference of single values ("residues") for each distance point and smoothed every ten points to improve readability. As an example, one dendrite of a EGFP/A-Ch expressing neurons treated with BDNF is straightened for clarity. Gray boxes represent areas of the figure that could not be reconstructed due to the original curvature of the dendrite. Scale bar, 10 µm. (**b**) Traces for A-Ch and S-Ch constructs following BDNF treatment. The residues for A-Ch are significantly higher than those calculated for S-Ch. Traces are single dendrites. (**c**) Plot values of residues for the two constructs as cumulative probability. Residues were sampled every 0.12µm along dendrites. ***$P<0.001$, Kolmogorov-Smirnov test.

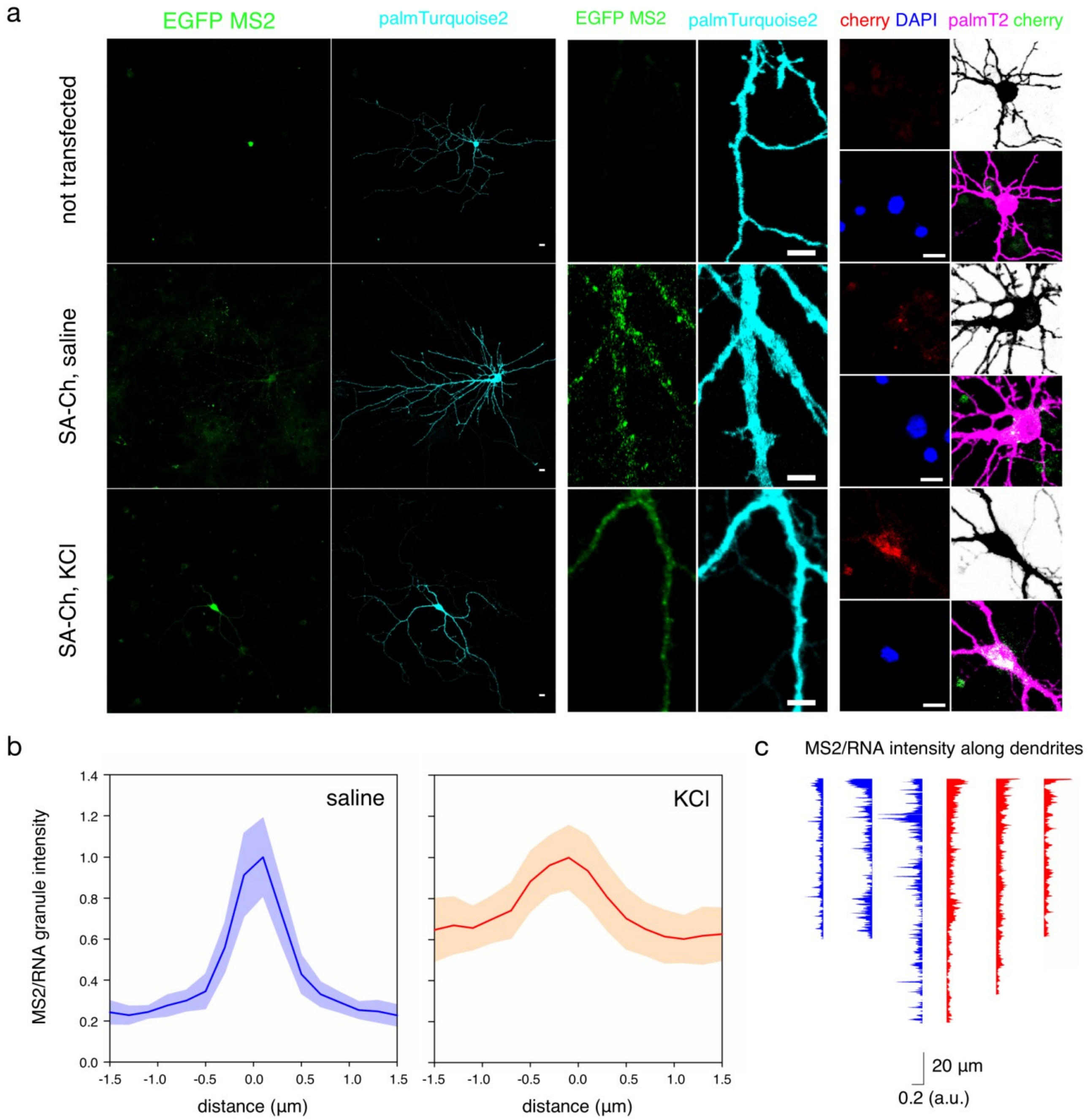

Supplementary Figure 4 SA-Ch transcript is present in granules along dendrites. (**a**) Confocal images of EGFP-MS2 (green), membrane-localized palmitoyl-Turquoise2 (palmT2, cyan in left and center panel), DAPI (blue) and ChETA-Cherry (red). SA-Ch RNA/MS2 is present in dendrites in a granule-like pattern in untreated neurons. Accordingly, a low, sparse, ChETA-Cherry signal is detected. Treatment with KCl releases mRNA from granules yielding a more diffuse signal, and an increase in ChETA-Cherry expression. Control cells expressing EGFP-MS2 alone show neither of the two signals and the signal is localized in the nucleus, due to the presence of NLS in the EGFP-MS2 protein. Please refer to Fig.1a for a scheme of the MS2 system. Scale bar, left and right panel 10 µm, central panel 5 µm. On the right column colours have been changed for consistency with main figures. (**b**) Average fluorescence intensity profile of RNA granules associated to spines. Under control conditions, granules are bright particles and nearby fluorescence is low (blue trace). KCl treatment induces granule disassembly and increase of MS2/RNA fluorescence in the surrounding region (red trace). Traces are 3 µm intensity profiles, centered at the brightest spot under synapses, after background subtraction and normalization to peak. Shadowed area is 2 standard errors from mean. (**c**) Intensity profile of MS2/RNA signal in representative dendrites in untreated (blue traces) and KCl-treated neurons (red traces). Signal is normalized to average intensity along dendrites.

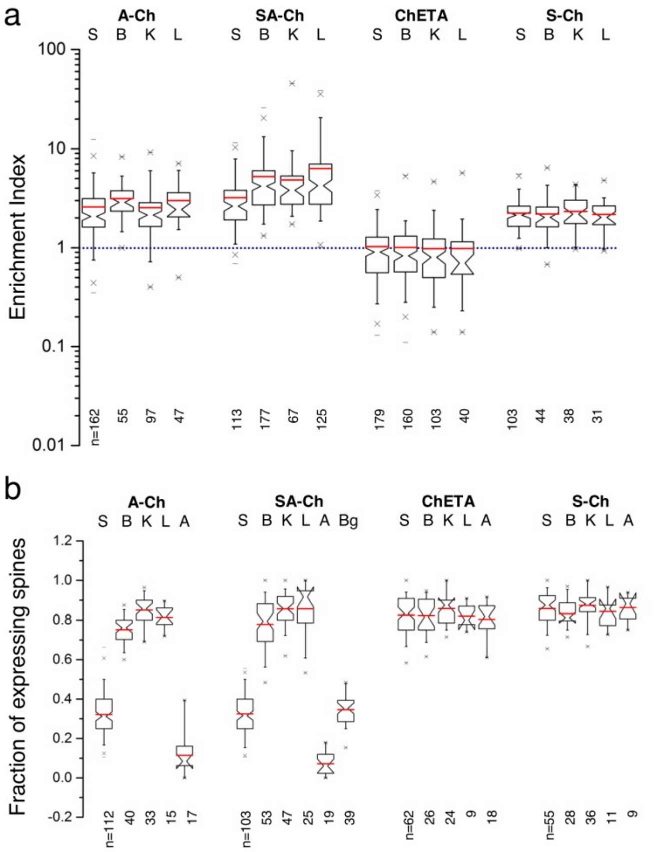

Supplementary Figure 5 Notched boxplots of data presented in Figure 1c (**a**) and 1d (**b**) in the main text. Notch is median ± 95% confidence interval of the median. Red line is mean, crosses are 1% and 99% of the distribution, horizontal lines are the corresponding extremes (minimum and maximum). Legend S:saline, B:BDNF, K:KCl, L:cLTP, A:AP5, Bg;BDNF+G418. Data are from 2 to 5 replicates each.

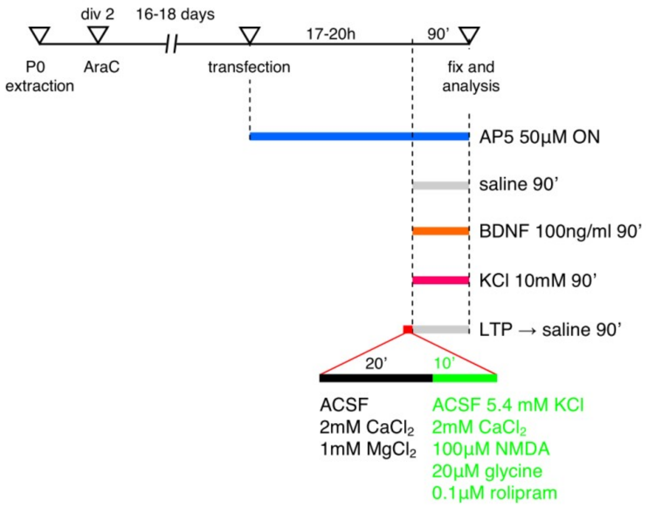

Supplementary Figure 6 Outline of experiments described in text. Div 17-19 neurons are used in every experiment unless otherwise stated.

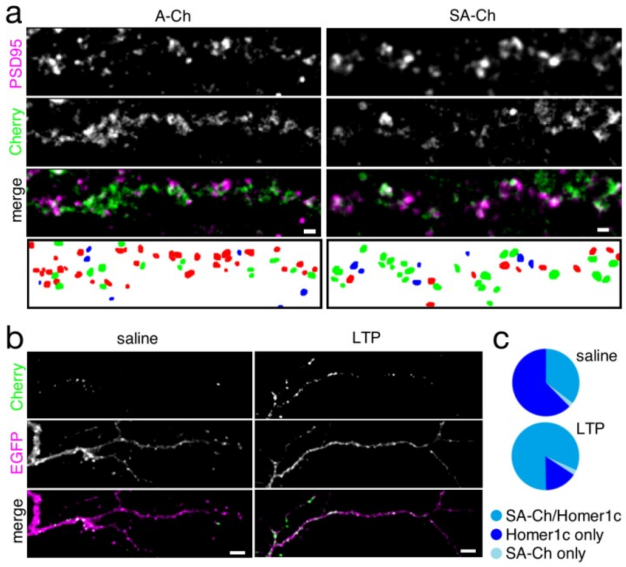

Supplementary Figure 7 (**a**) Another image of SA-Ch and A-Ch expressing neurons stained for PSD95 (magenta in merge) and Cherry (green in merge) IF. Bottom panel: docked synapses (green), positive, non-docked synapses (red), Cherry-negative synapses (blue). See text and Figure 2a for definition of "docked" spine. Scale bar 1 µm. (**b**) Spine-specific localization pattern of SA-Ch in hippocampal cultures following saline or LTP treatment. SA-Ch pattern (cherry) largely overlaps with Homer1c-EGFP accumulation puncta marking postsynaptic densities. Scale bar 5 µm. (**c**) Quantification of Homer1c-EGFP puncta that were positive for SA-Ch following saline or LTP treatment. A very small fraction of SA-Ch points were not evidently associated with corresponding Homer1c-EGFP signal.

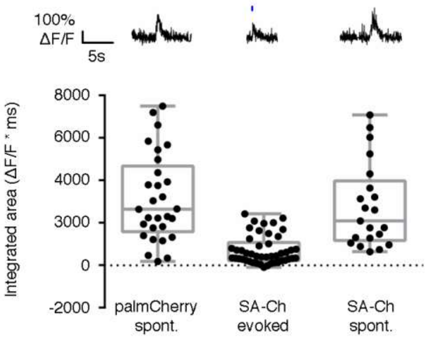

Supplementary Figure 8 Spontaneous calcium transients from palmitoyl-Cherry expressing cells are not different from spontaneous events registered from SA-Ch expressing spines from responsive neurons. SA-Ch evoked events are single traces from responsive, light-evoked events represented in Figure 4a in the main text. Top, representative traces of spontaneous transients (traces were cropped for clarity) from neurons expressing palmitoyl-Cherry (left) or SA-Ch (right). Center, light-evoked calcium transient of a single-trace recording from the same spine represented on the right.

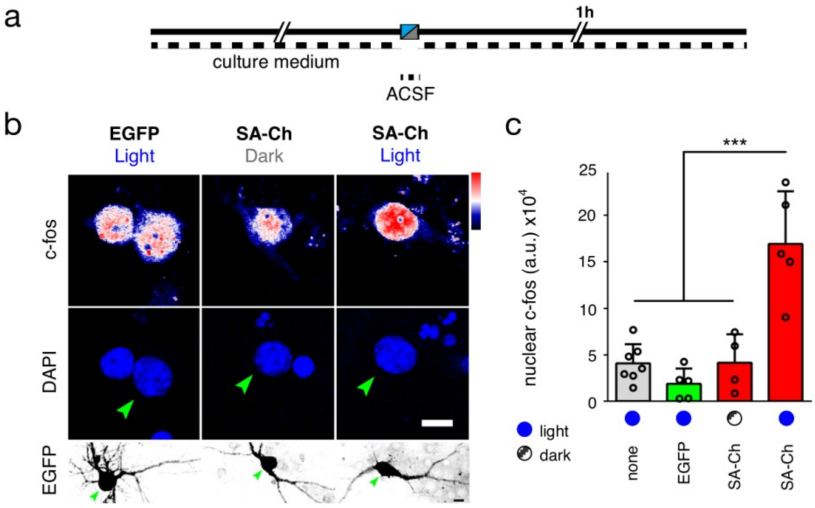

Supplementary Figure 9 (**a**) Outline of time course of the experiment. Cells were fixed and stained for c-fos 60 minutes after light stimulation. (**b**) c-fos (top) and DAPI (middle row) staining of cells expressing EGFP (left) or SA-Ch and EGFP (middle and right). Cells were illuminated or maintained in the dark as indicated above. Green arrowheads indicate corresponding positions in the EGFP channel below. Scale bar, 5 µm. (**c**) Nuclear c-fos staining for illuminated, EGFP expressing neurons, and SA-Ch/EGFP neurons maintained in the dark is comparable to untransfected cells. Optical stimulation of SA-Ch/EGFP neurons increases c-fos expression in the nucleus. ***P<0.001, one-way ANOVA, Bonferroni comparison of means. Bars are mean±s.d.

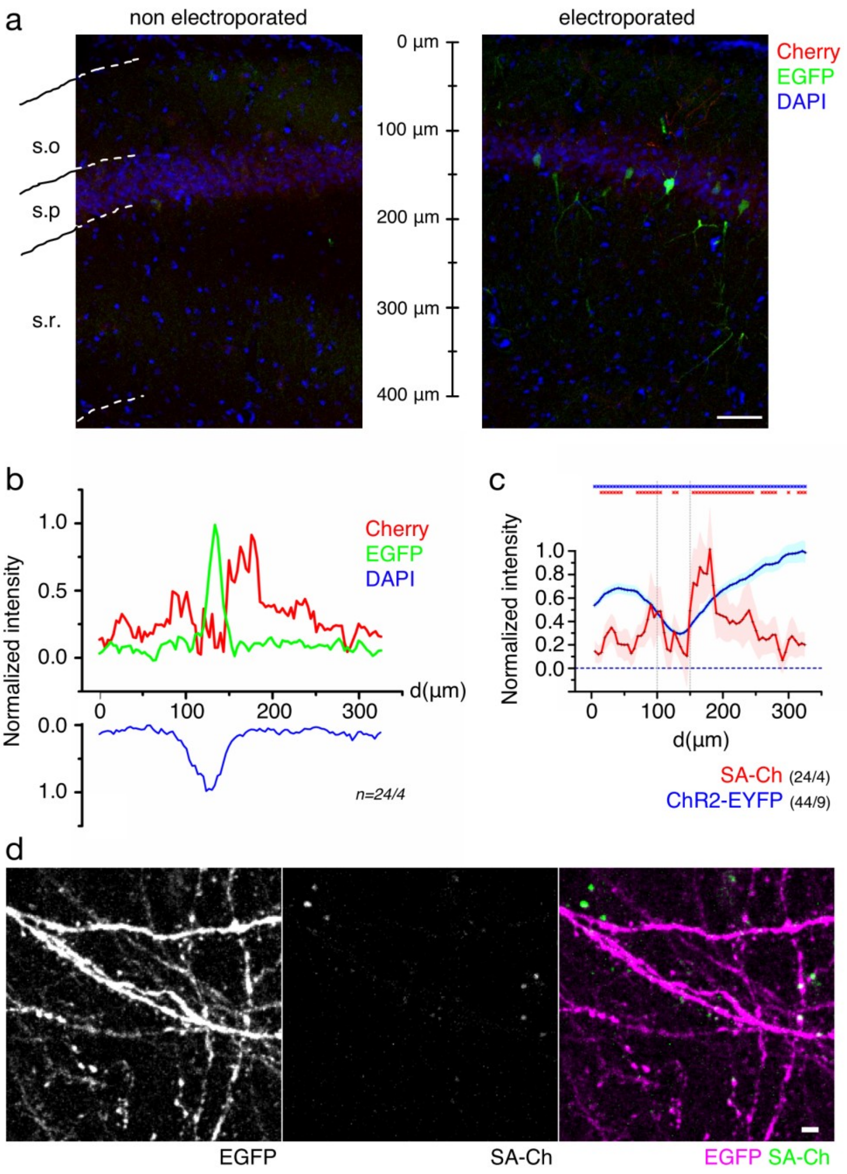

Supplementary Figure 10 Expression of SA-Ch in mouse hippocampus. (**a**) CA1 region comprising the stratum oriens (s.o.), the stratum pyramidale (s.p.) and the stratum radiatum (s.r.) from a mouse unilaterally electroporated with TRE:SA-Ch and TRE:EGFP. Right: electroporated hemisphere and left: control hemisphere from the same slice. EGFP is in green, Cherry in red and nuclei are stained with DAPI, scale bar 50µm. Profiles were plotted along radially oriented lines starting from the stratum oriens. (**b**) Line plot the average of 24 profiles from 4 animals, after subtracting the baseline, which was calculated in the non-electroporated hemispheres. The majority of EGFP signal (green line) is concentrated in the soma, in correspondence to the DAPI signal (blue line). In contrast, SA-Ch was most abundantly expressed in the dendrites in the stratum oriens and in the stratum radiatum. (**c**) Profile of SA-Ch expression (red) compared to untargeted Channelrhodopsin from Thy1:ChR2-YFP mice (blue). For every trace, values were averaged every 5µm starting from the beginning; for each construct, we plot the average of the corresponding profiles (line and cross)±s.e.m. (shadowed areas). Numbers in parentheses indicate the number of profiles/slices for each sample. Plots were normalized on the highest value. The two constructs are significantly different at the α=0.001 level (two-way ANOVA). Asterisks on the top indicate distance points that are significantly different from zero (dashed line) for SA-Ch (red) and ChR2-YFP (blue). Untargeted ChR2-YFP, but not SA-Ch, is significantly different from zero in the 100-150µm range (z-test, α=0.05). (**d**) SA-Ch expression in dendrites in CA1 stratum radiatum in a home caged mouse after 3.5 days of doxycycline administration. Scale bar, 2µm.

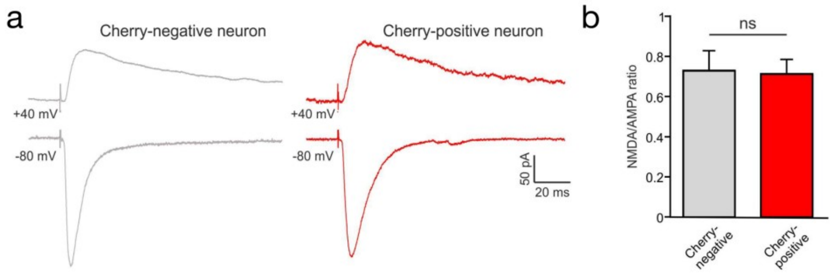

Supplementary Figure 11 SA-Ch expression does not affect the NMDA/AMPA ratio at CA3-CA1 synapses. . (**a**) Representative traces of isolated AMPA- (bottom) and NMDA- (top) EPSCs evoked by Schaffer collateral stimulation in one Cherry-negative CA1 cell (grey traces) and one Cherry-positive CA1 neuron (red traces). Mice were electroporated with TRE:SA-Ch and CAGG:rtTA-IRES-mCherry and induced for 4 days with 0.5mg/day i.p. doxycycline as previously. AMPA-EPSCs were recorded at V_m = -80 mV, NMDA-EPSCs at V_m = +40 mV. The average of ten traces is shown. Stimulation artefacts have been truncated for presentation purposes. (**b**) NMDA/AMPA ratio for Cherry-negative (n=7 cells from 4 mice) and Cherry-positive (n= 9 cells from 5 mice) neurons. Average values are expressed as mean±s.e.m. The Mann-Whitney test was used for statistical comparison, P = 0.52. ns (non-significant).

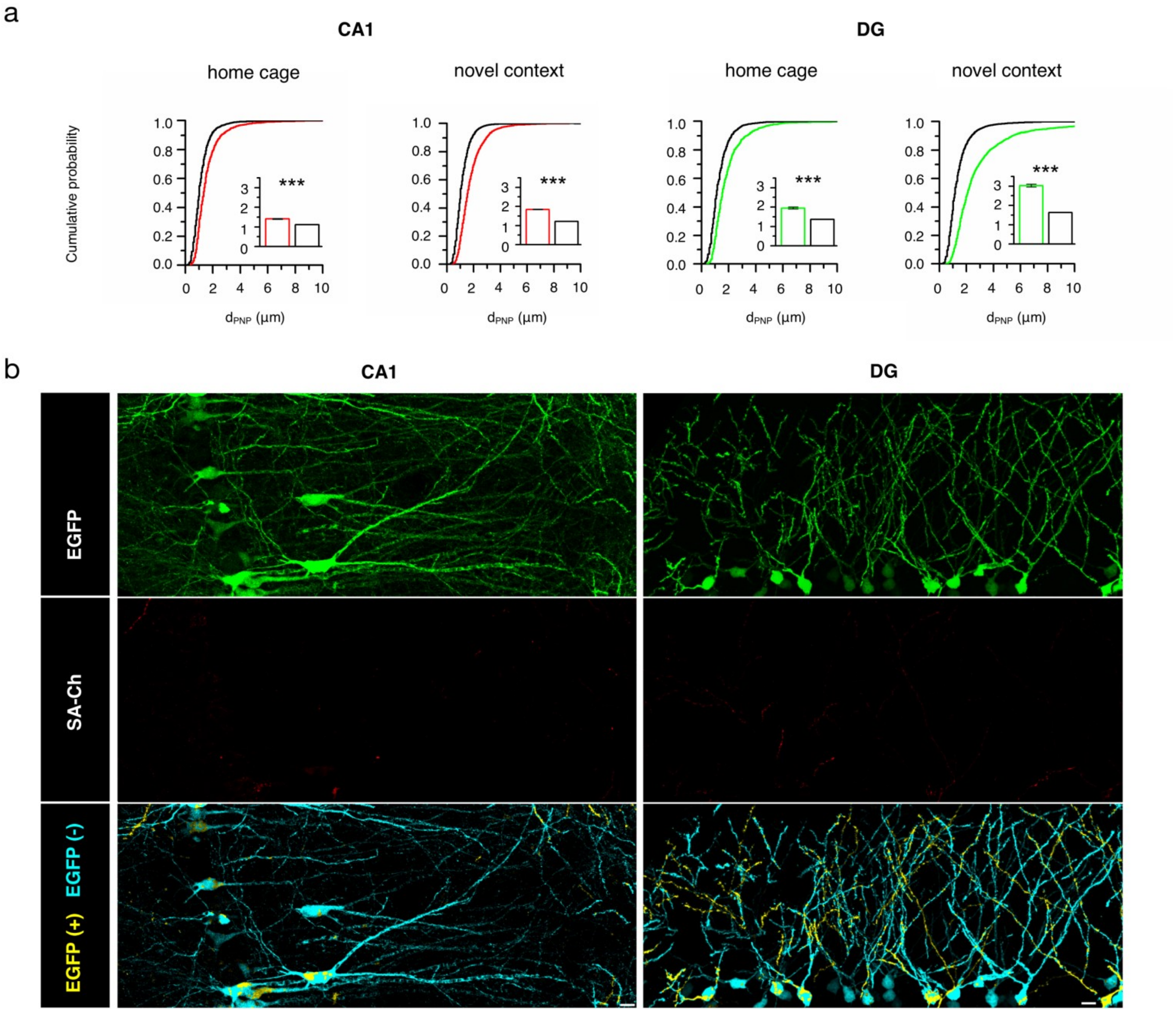

Supplementary Figure 12 (**a**) Distribution of distances to first non potentiated neighbour (d_{PNP}) in CA1 (red) and DG (green) in home cage and novel context groups. Black lines represent the distribution for the values obtained with randomly shuffled positions. Insets are mean±s.e.m. ****P*<0.001, Kruskal-Wallis test, Dunn's comparison. (**b**) Original image depicted in Figure 5g in the main text. EGFP(+) is the region of the cells that express SA-Ch (yellow), whereas EGFP(-) is the complementary region (cyan). To generate EGFP(+) image, the Cherry channel was thresholded to remove background, and the resulting mask was expanded for clarity with "dilate" command in ImageJ.

Supplementary Methods DTEs and ATE used in the study

Arc DTE

Arc DTE maps nucleotides 2035-2702 of Author's sequence (Kobayashi et al, Eur J Neurosc, 2005). See as reference NCBI entry NM_019361.1 [Rattus norvegicus activity-regulated cytoskeleton-associated protein (Arc), mRNA] with T2130A mismatch and T2293Δ deletion, as reported by the Authors (H.Kobayashi, personal communication).

TTCGGCTCCATGACTCAGCCATGCCAGGCGGAGGGTCCCAGAGGGGCTGAGTCCTCAGCCCCAGCTGAGGCAGCAGC
TGGAGTCTTCAGAGCCAGGAGAATGACACCAGGTCTCAAGCTGCTGAGAAGTCTTTCCGGCCATGTCTGGAAGGGGTA
CCACCCCAGCACCAGCACCGTCCCCTCCTCTCTTGAAGCTGCCTGCACAGAGGTTCCAAGACACTTTCAAGGCAGAGA
AAATAGGATTACAAAGAGGAGGTGCCTGGCAGAGGGCAGCACCCAGCTCAGCCTCAGAGCTGAAGGTGAAGACAAG
CCAGCGTGAAACCCCGGGTCTGCCACGAATGCCCGCTCCGCTGGCCACTCACCAGCTGCCTGCCACAAGCCACTGCAG
CTTGAGCAGGGTCTGTGCCCTCTCAGCACAGAGCCCAGTTCGCTGCGTGGCCTTTGGCCCCCGCCAGAACCTTGCAGG
AGCCTTAAGGTTCGGGCCCTAGCCCAGCCTGACCTTACCTGCTGTGCCCTGCCTGCTGGTCAAGTCCAGTCCCAGGAG
ACCCCATGCCTTGGCTCCTAGGCTGTTCCAGGCACTTCCCTGACCTGCCGGGTGATTGCCCAGCTGGAACCTCATCCAC
ACCCCAGCACCAACCACCTCGTGTTGGTAACTGCTCGTGTCTGTA

CaMKII DTE

Sequence cloned in pNECKu1481-2708 in (Blichenberg et al, Eur J Neurosc, 2001).

GATCCCTTGTCTGCACTGTTTTCTTTGCATGACTTTATATGCAGTAAGTATGTTGAGAAAAAAAAGAGCAAAGACAAA
AAAGAAGAAAAACACTCAGCAAAATCAAACGACACGTTTTGGACAAAAAATATAATAATAACATTCAAGGTTATATT
CTCAGTGTCCAACTTGGAATTACGTTGCTGCCTCTCTGTGCTTTTGGTCTCTGTGTGGCTGTGTTTTGCCAGCATGAGAC
CCTGTCCCCTCTGGAGGTTTCTAGGGGAGGAAGAGCCGTGTGTCGGGGGGGGGGGTTGGAGACAGCTTTGTCCTCTCA
GCTTTTTGGGGGGGTTGATTGGAGCAGAAGTGGAAGGGGATGTTTAATCCAGAACTTTCTGGTATTTCCCTTTCTCCCA
CGCAGTGAGCTATACGCTGGGCTCTTCTCTCAAATCCTGCTGCCCAGGGACAAGTATAGGGTAGAAGGGTGGCCCTAT
TGTCTAAGCCACTCCACTGTAGCCCTCTGCCTTTGGTAGAGACACTGCTACCCAGACCCAAGAATGGGCCCTTGTCCCA
CCCCAGATCTAGGCTTCTTCATAAGGCTCAGCAAACTCATTGTCCCCAGCCATTCCCCCACTAAAGGTAAAAGAAGGT
GTGGCCTTTACCAGGGGACACTGCGATTATCAATCAAGCCCTCTTCAAGCCTCAGTTTCACCACCAATGTTCCTACCCA
GACTGATGGAAGGTCAAACTAAATGATGTCACAAGTGCACACCATCTTTGAGAACTTGCTGGGTTTGTCACTGGCTGG
CCTTCTTATGCACCAGGCCCGGCCAATTCCCATCTTTTCCCCTGTGTGCCCCCTCATTTTCCTATTTGGTGCCAGTCTGT
TGAAGACCAGCAACAAATGCAGGGGAAAGAAGTGTCTGGGGGCTTTGGTAGGCTTTGACCCCCCGTTCTGATCAGAA
GGGCTGTGTGGCTTTGGGTGAGTCCTGTGCCCTCCTGGGGCCTTAGTTTCCTCAGCCAGAAGATGCCTATGCCCTGCCT
TCTGTTGGCTAACATGCCCCTGTCCACTGTGTGCCTGTCCACATGTGGAGAAGTGGAGGCAGGTCCCTGCCCCAGTCTG
AGACGGCCCGCTCTGCAGAGGCCGCTCCTGTGGGTGGGCAGCCAACTCATGTAGACCTTGGGACACTACAATGGCCCC
AAGGTAGCAGGCAGGGGAACTGGCAGAAAAACTGCCCTCCTCAGACAAGCT

MAP2 DTE

Sequence cloned in pNEu2432-3071 in (Blichenberg et al, *J Neurosc*, 1999).

AGCGGCCGCGATCTAGCACTAAAATATCATTTTTCTAATGTTAATACAATTATAATGGATACAAGTCCTTGTTTTATGT
GAAAATGTGATTCACACATGAATGTAAAGTCAACACAAGAAGGACCTGAATTTTTTGTACCAGACAGAGACAGAGAA
ATGCACAGGCTAAAATTCACTTCCTTATGGGAATGTGGGATGGATCCCACCTTACCTACTTAAGATAATGACTCAAAT
TAAGCTTTTTGGACACCACTTTTGTGGGGATACACATACGCTGATCTAGAAATGAAAGGCGCACAGCTACATTTCTAG
ATCCACTAATGCCAGTTTCTCTTTGGCTTCAGCCTTTGAGAACCTGTTCAAGAATACGTAAGTATCCAGAGCTCTGAAG
AGTTCGAAGGCCAACTTTTTCAGTGAACTCACACACTCTGGGTCTCCTGCAACTGACAATTGGGTACCTTGCAACAAT
GCGGGAAGGATCCGAGTTTATGATGAGTTTCAAAGGCCGTGTTCACTTAGGAACTGACTCTCTCTGGATCTGCCTGCT
GCGTTCCAGCAGGATGACGGGCTGAAATCCCACCCATAGGGAAGACACCTGTGCAATTCCAGCTCAGTTTGGCTGAAG
GTAACTAAAGAAGAAGGTCCAGTAA

IMPA1 ATE

Sequence cloned in pSC-A IMPA1L (Andreassi et al, *Nat Neurosc*, **13**, 291--301, 2010) corresponding to IMPA1 nts 2044-2165. Sequence maps nts 1126-1249 of NCBI entry GU441530.1 [Rattus norvegicus strain Sprague-Dawley inositol (myo)-1(or 4)-monophosphatase 1 (Impa1-L) mRNA, 3' UTR]

CTGTATTTATGCTGCTAATTACATGCATTTAAAACATCAGGAACCATGTAAATCCTATTACAAGACAGGTTGCTTTTGC
AATTAAATTTATTTACTTACAAGC

	sample	Min/25%/median/50%/Max	n	replicates	P value
Fig 2b	A-Ch	0.302/0.426/0.447/0.488/0.547	11	2	A-Ch vs. SA.Ch unpaired Student's t-test, two-tailed<br>2.36365E-21
	SA-Ch	0.677/0.786/0.833/0.867/0.935	33	2	
Fig 2c	A-Ch	0.863/0.901/0.914/0.931/0.953	11	2	A-Ch vs. SA.Ch unpaired Student's t-test, two-tailed<br>0.97014354
	SA-Ch	0.8/0.891/0.92/0.943/0.987	33	2	
Fig 2e	SA-Ch untreated	NA	290 (27 neurons)	5	Linear regression of Log(values)<br>untreated slope 1.892±0.086 df_n=1 df_d=228 (without SA-Ch EI = 0 points)<br>untreated slope 2.642±0.137 df_n=1 df_d=286 (Log(SA-Ch EI) was assigned value -2 for SA-Ch EI=0 points)<br>stimulated slope 2.290±0.167 df_n=1 df_d=77
	SA-Ch stimulated	NA	79 (10 neurons)	2	
Fig 2g	SA-Ch	NA	468 (71 neurons)	6	Linear regression of Log(values)<br>SA-Ch slope 0.9749±0.03459 df_n=1 df_d=396 (without SA-Ch EI = 0 points)<br>S-Ch slope 0.2150±0.02447 df_n=1 df_d=267
	S-Ch	NA	269 (37 neurons)	3	
Fig 3b	SA-Ch, MNI+fsk; s	0.365/1.029/3.047/4.558/11.35	25	9	One-way ANOVA 2.09832E-14, Bonferroni comparison of means:<br>SA MNI+fsk s vs. SA MNI+fsk n 5.48E-15<br>SA MNI+fsk s vs. SA fsk s 1.20761E-9<br>SA MNI+fsk s vs. SA fsk n 1.23116E-10<br>SA MNI+fsk s vs. S MNI+fsk s 4.81463E-4<br>SA MNI+fsk s vs. S MNI+fsk n 1.20215E-5<br><br>S MNI+fsk s vs. S MNI+fsk n : unpaired Student's t-test, two-tailed, Welch's correction 3.78818E-4
	SA-Ch, MNI+fsk; n	-0.834/-0.46/-0.105/0.523/2.128	46		
	SA-Ch, fsk; s	-0.69/-0.358/-0.11/0.417/1.821	15	4	
	SA-Ch, fsk; n	-0.738/-0.35/0.096/0.349/1.583	18		
	S-Ch, MNI+fsk; s	0.496/0.728/1.044/1.181/1.268	8	3	
	S-Ch, MNI+fsk; n	-0.366/-0.348/0.072/0.362/0.436	6		
Fig 3d ΔV/V	MNI+fsk; s	NA	22	8	NA
	MNI+fsk; n	NA	24		
	MNI+fsk+anys; s	NA	15	4	
	MNI+fsk+anys; n	NA	21		
	MNI; s	NA	11	4	
	MNI; n	NA	8		
Fig 3d ΔCh/Ch	MNI+fsk; s	NA	18	6	NA
	MNI+fsk; n	NA	24		
	MNI+fsk+anys; s	NA	15	4	
	MNI+fsk+anys; n	NA	21		
	MNI; s	NA	8	3	
	MNI; n	NA	8		
Fig 4a	ACSF	-6.563/261.088/446.831/744.326/1018.398	21	7	Kruskall-Wallis test of one-way ANOVA, Dunn's test<br>ACSF vs. ACSF no stim 0.0036<br>ACSF vs. VGCC inh 0.0003<br>ACSF vs. VGCC inh no stim 0.0064<br>ACSF vs. TTX >0.999<br>TTX vs. TTX no stim 0.0053
	ACSF no stim	-255.74/-185.019/-58.183/119.138/149.746	8	3	
	VGCC inh	-91.493/-19.504/57.271/82.74/117.5	17	4	
	VGCC inh no stim	-368.323/-210.356/53.643/103.581/152.094	8	2	
	TTX	-26.478/214.382/670.663/889.896/1154.409	17	4	
	TTX no stim	-243.011/-147.721/-116.593/267.253/282.191	7	3	
Fig 4b	SA-Ch spine	86.987/200.365/690.225/1139.499/2501.239	10	4	Paired Student's t-test, two-tailed<br>SA-Ch spine vs. dendrite t=3.686 df=9 P=0.005<br>ChETA spine vs. dendrite t=0.4454 df=10 P=0.6655
	SA-Ch dendrite	-205.586/-38.087/-4.111/63.192/261.866			
	ChETA spine	216.865/242.926/534.5/1236/1887.415	11	4	
	ChETA dendrite	231.715/400.722/624.056/883.32/1391.364			
Fig 4d	EGFP light	45.792/1806.92/2951.662/4627.827/21196.312	364	2	One-way ANOVA, Bonferroni comparison of means<br>SA-Ch light Ch+ vs. EGFP light 2.63728E-143<br>SA-Ch light Ch+ vs. EGFP dark 2.10596E-140<br>SA-Ch light Ch+ vs. SA-Ch light Ch- 2.39868E-182<br>SA-Ch light Ch+ vs. SA-Ch dark Ch+ 3.53488E-205<br>SA-Ch light Ch+ vs. SA-Ch dark Ch- 2.05879E-186<br>SA-Ch light Ch+ vs. ChETA light 2.42246E-104<br>SA-Ch light Ch+ vs. ChETA dark 6.02358E-233<br>SA-Ch light Ch- vs. EGFP light >0.999<br>SA-Ch dark Ch+ vs. EGFP dark >0.999<br>ChETA light vs. ChETA dark 3.77679E-34
	EGFP dark	53.092/1747.77/3088.081/4913.416/27877.427	347	2	
	SA-Ch light Ch+	34.134/5676.424/9672.529/15828.503/91633.554	1051	2	
	SA-Ch light Ch-	-111.551/1931.315/3200.99/4871.958/27607.742	539		
	SA-Ch dark Ch+	-5.994/2007.332/3432.829/5711.051/23357.363	751	2	
	SA-Ch dark Ch-	21.232/2049.816/3319.291/5116.423/19137.313	557		
	ChETA light	530.912/4155.527/6285.748/9236.164/36990.65	1002	2	
	ChETA dark	10.808/2088.084/3329.345/5109.629/20177.826	890	2	
Fig 5b	CA1 hc	0.0476/0.1277/0.1702/0.2167/0.573	93	3	Unpaired Student's t-test, two-tailed, Welch's correction<br>CA1 hc vs. CA1 cnt 8.06341E-13<br>DG hc vs. DG cnt 0.02098
	CA1 cnt	0.037/0.2055/0.3107/0.48/0.8529	111	3	
	DG hc	0.037/0.139/0.2623/0.3869/0.6415	52	3	
	DG cnt	0.0588/0.1346/0.2705/0.5636/0.9074	58	3	
Fig 5d Fig S12a	CA1 hc dPP	0.214/0.688/0.999/1.711/32.181	1172	3	Kruskal-Wallis test, Dunn's comparisons<br>mean rank differences:<br>CA1 hc dPP vs. CA1 hc dPP shuffled -2875<br>CA1 hc dPNP vs. CA1 hc dPNP shuffled 1711<br>CA1 cnt dPP vs. CA1 cnt dPP shuffled -8137<br>CA1 cnt dPNP vs. CA1 cnt dPNP shuffled 9785<br>DG hc dPP vs. DG hc dPP shuffled -2796<br>DG hc dPNP vs. DG hc dPNP shuffled 2244<br>DG cnt dPP vs. DG cnt dPP shuffled -9048<br>DG cnt dPNP vs. DG cnt dPNP shuffled 4923
	CA1 hc dPNP	0.329/0.955/1.236/1.608/8.747	1172		
	CA1 cnt dPP	0.214/0.688/0.906/1.179/15.7	3474	3	
	CA1 cnt dPNP	0.392/1.179/1.596/2.188/11.2	3474		
	DG hc dPP	0.151/0.755/1.068/1.604/26.6	1211	3	
	DG hc dPNP	0.338/1.117/1.546/2.218/28.03	1211		
	DG cnt dPP	0.302/0.755/0.967/1.281/19.54	1886	3	
	DG cnt dPNP	0.338/1.478/2.092/3.276/25.91	1886		
Fig 5e	CA1 hc	NA	91	4	NA
	CA1 cnt	NA	108	3	
	DG hc	NA	49	4	
	DG cnt	NA	53	3	
Fig 5f	CA1 hc	1/1/2/3/25	630	4	Kruskal-Wallis test, Dunn's comparisons<br>mean rank differences<br>CA1 hc vs. CA1 cnt -360
	CA1 cnt	1/2/3/6/29	859	3	
	DG hc	1/1/2/4/26	334	4	

	DG cnt	1/2/3/6/29	450	3	DG hc vs. DG cnt -275.9
Fig 5h	CA1 hc	0/0.13/0.297/0.527/1.822	93	4	Kruskal-Wallis test, Dunn's comparisons mean rank differences CA1 hc vs. DG hc -28.21 CA1 cnt vs. DG cnt –52.6
	CA1 cnt	0.001/0.178/0.412/0.587/1.257	111	3	
	DG hc	0.038/0.196/0.42/0.643/1.288	52	4	
	DG cnt	0.015/0.415/0.6/0.736/1.19	58	3	
Fig S1	CaMKII	0.021/0.408/0.531/0.685/0.805	46	2	One-way ANOVA, Bonferroni comparison of means Arc vs. CaMKII 0.00694 Arc vs. CaMKII KCl 7.50859E-7 Arc vs. MAP2 4.35833E-6 Arc vs. Arc KCl 8.16394E-36 Arc vs. EGFP 1.97554E-21 Arc KCl vs. CaMKII KCl 0.00588
	CaMKII KCl	0.045/0.47/0.631/0.828/1.308	32	2	
	MAP2	0.086/0.371/0.525/0.675/1.056	121	2	
	Arc	-0.271/0.189/0.335/0.497/0.906	173	2	
	Arc KCl	0.37/0.72/0.924/1.001/.415	77	2	
	IMPA1	-1.925/-0.473/-0.236/-0.428/0.459	177	2	
	EGFP	-1.67/-0.165/0.045/0.267/0.909	169	2	
Fig S2b	EGFP	0.31/0.486/0.575/0.66/1.789	33	2	One-way ANOVA, Bonferroni comparison of means P>0.999 for all pairwise comparisons
	ChETA	0.269/0.495/0.624/0.686/0.948	36	2	
	S-Ch	0.413/0.518/0.603/0.648/0.951	34	2	
	SA-Ch	0.384/0.535/0.595/0.641/0.825	27	2	
Fig S2c	EGFP s	0.075/0.125/0.186/0.241/0.556	33	2	Two-way ANOVA, Factor A Construct DF=3 P<0.0001 Factor B Spine type DF =2 P>0.999 Interaction DF=6 P=0.6605
	EGFP m	0.267/0.417/0.522/0.562/0.678			
	EGFP t	0.111/0.205/0.314/0.393/0.489			
	ChETA s	0.071/0.150/0.227/0.286/0.412	36	2	
	ChETA m	0.344/0.422/0.491/0.593/0.688			
	ChETA t	0.125/0.234/0.28/0.323/0.438			
	S-Ch s	0.079/0.178/0.218/0.267/0.378	34	2	
	S-Ch m	0.216/0.425/0.509/0.556/0.681			
	S-Ch t	0.191/0.244/0.289/0.336/0.448			
	SA-Ch s	0.115/.159/0.209/0.243/0.3	27	2	
	SA-Ch m	0.324/0.423/0.514/0.577/0.667			
	SA-Ch t	0.133/0.226/0.281/0.367/0.467			
Fig S2d	SA-Ch	0.0531/0.2212/0.3542/0.5169/0.7574	52	3	Unapired Student's t-test two-tailed, P=0.609
	pTurquoise2	0.0939/0.1921/0.2943/0.4946/1.046	54	4	
Fig S3	A-Ch BDNF	NA	5	1	Kolmogorov-Smirnov test, P<0.001
	S-Ch BDNF	NA	15	1	
Fig S4b	Saline	NA	15	1	NA
	KCl	NA	15	1	
Sig S7	Saline	NA	28	2	NA
	LTP	NA	20	2	
Fig S8	EGFP light	NA	5	2	One-way ANOVA, Bonferroni comparison of means SA-Ch light vs. EGFP light 1.13809E-5 SA-Ch light vs. NT light 3.19138E-5 SA-Ch light vs. SA-Ch dark 1.716E-4
	NT dark	NA	7	2	
	SA-Ch dark	NA	4	2	
	SA-Ch light	NA	5	2	
Fig S9	pCherry spont	181/1578/2638/4670/7494	29	2	Kruskal-Wallis test, Dunn's comparison SA-Ch evoked vs. pCherry spont P<0.001 SA-Ch spont vs. pCherry spont P>0.999
	SA-Ch evoked	-96.66/224/435.9/1072/2423	46	7	
	SA-Ch spont	641.3/1163/2086/3970/7082	21	3	
Fig S10	SA-Ch	NA	24	4	Two-way ANOVA Factor A construct DF=1 P<0.0001 Factor B distance DF=64 P<0.0001
	Thy1-ChR2	NA	44	2	
Fig S11	Cherry +	50/53.5/67/80/120	9	5	Mann-Whitey test, two tailed P=0.5163
	Cherry -	30/51/78/87/112	7	4	

Supplementary Table 1 Statistical information for data presented in the main text and in the Supporting Information. NA = not applicable
